# Protective effect of low‐intensity pulsed ultrasound on immune checkpoint inhibitor-related myocarditis via fine-tuning CD4^+^ T-cell differentiation

**DOI:** 10.1007/s00262-023-03590-5

**Published:** 2024-01-18

**Authors:** Shuai Fu, Zihong Guo, Xiangli Xu, Yifei Li, Stephen Choi, Peng Zhao, Wenqian Shen, Fei Gao, Chao Wang, Shuang Chen, You Li, Jiawei Tian, Ping Sun

**Affiliations:** 1 Department of Ultrasound, The Second Affiliated Hospital of Harbin Medical University, Harbin, Heilongjiang Province China; 2 Ultrasound Molecular Imaging Joint Laboratory of Heilongjiang Province, Harbin, Heilongjiang Province China; 3 The Key Laboratory of Myocardial Ischemia, Harbin Medical University, Ministry of Education, Harbin, Heilongjiang Province China; 4 Department of Ultrasound, The Second Hospital of Harbin, Harbin, Heilongjiang Province China; 5 SXULTRASONIC Ltd. Kerry Rehabilitation Medicine Research Institute, Shenzhen, Guangdong Province China

**Keywords:** Immune checkpoint inhibitors, Programmed death 1, Myocarditis, Low-intensity pulsed ultrasound, HIPPO

## Abstract

**Purpose:**

Immune checkpoint inhibitors (ICIs) have transformed traditional cancer treatments. Specifically, ICI-related myocarditis is an immune-related adverse event (irAE) with high mortality. ICIs activate CD4^+^ T-lymphocyte reprogramming, causing an imbalance between Th17 and Treg cell differentiation, ultimately leading to myocardial inflammatory damage. Low-intensity pulsed ultrasound (LIPUS) can limit inflammatory responses, with positive therapeutic effects across various cardiovascular inflammatory diseases; however, its role in the pathogenesis of ICI-related myocarditis and CD4^+^ T-cell dysfunction remains unclear. Accordingly, this study investigated whether LIPUS can alleviate ICI-related myocarditis inflammatory damage and, if so, aimed to elucidate the beneficial effects of LIPUS and its underlying molecular mechanisms.

**Methods:**

An in vivo model of ICI-related myocarditis was obtained by intraperitonially injecting male A/J mice with an InVivoPlus anti-mouse PD-1 inhibitor. LIPUS treatment was performed via an ultrasound-guided application to the heart via the chest wall. The echocardiographic parameters were observed and cardiac function was assessed using an in vivo imaging system. The expression of core components of the HIPPO pathway was analyzed via western blotting.

**Results:**

LIPUS treatment reduced cardiac immune responses and inflammatory cardiac injury. Further, LIPUS treatment alleviated the inflammatory response in mice with ICI-related myocarditis. Mechanistically, in the HIPPO pathway, the activation of Mst1–TAZ axis improved autoimmune inflammation by altering the interaction between the transcription factors FOXP3 and RORγt and regulating the differentiation of Treg and Th17 cells.

**Conclusion:**

LIPUS therapy was shown to reduce ICI-related myocarditis inflammatory damage and improve cardiac function, representing an exciting finding for irAEs treatment.

**Supplementary Information:**

The online version contains supplementary material available at 10.1007/s00262-023-03590-5.

## Introduction

Immune checkpoint inhibitors (ICIs) are monoclonal antibodies that target and block immune checkpoint functions. They prevent shutdown signaling to immune cells, stimulate downstream signaling pathways to enhance T-cell proliferation and differentiation, prevent tumor immune escape, and subsequently mediate anti-tumor activity [1, 2]. Over the past decade, ICIs have been approved as a first line therapy for more than one dozen malignancies, and this number is expected to increase in coming years [3] as ICI-based therapies have revolutionized cancer treatment [2].

While enhancing antitumor effects, an overactivated immune system can damage normal bodily tissues and organs due to increased T-cell activation, causing inflammatory side effects known as immune-related adverse events (irAEs) [4]. Among them, myocarditis often leads to fatality [5, 6]. Despite the low incidence of cardiotoxicity, poor prognosis and a lack of specificity of clinical presentation are a cause for concern in the medical field [7, 8]. Although some irAEs are self-limiting, fatal events have been reported [9]. However, the treatments recommended by various guidelines are mainly summarized from the treatment experience of non-ICI-related cardiotoxicity [9]. The most widespread recommendation for treatment is high-dose corticosteroids, without specificity, leading to poor cardiac outcomes and drug-resistance events [10, 11]. Other immunosuppressive therapies mainly targeting T-lymphocytes, including abatacept and Janus kinase (JAK) inhibitors, are being investigated [12]; thus, the interaction between tumor immunotherapy and cardiovascular system side effects becomes a concern, and new management strategies for ICI-related myocarditis are urgently needed [2, 13].

Pathological mechanisms of local inflammation and injury have been demonstrated to result from the infiltration of activated T-lymphocytes (primarily CD4^+^ and CD8^+^ T-cells) into cardiomyocytes, increasing the release of inflammatory factors after ICIs [6]. In preclinical studies, mice in which PD-1 has been genetically deleted have shown evidence of T-lymphocyte accumulation in the heart and immune-mediated myocardial disease, underscoring a critical role for immune checkpoints regulating T-cells in the heart [14]. Furthermore, multiple previous studies have linked activated effector CD4^+^ T-cells to fatal myocarditis [15, 16]. CD4^+^ T-cells undergo differential polarization after activation. Th17 cells are vital in autoimmunity and tissue injury, and Treg cells are essential for mediating immune tolerance and immunosuppression [17]. Contrastingly, the imbalance between Treg and Th17 cells represents an important factor in cancer immune escape and irAEs [14]. As their lineages are interconnected, modulating the balance of their correlated cellular differentiation is a promising method for improving irAE-related myocarditis.

Low-intensity pulsed ultrasound (LIPUS) emits pulsed sound waves, with relatively lower intensity than conventional ultrasound, to lesions and produces therapeutic effects through acoustic radiation force [18]. A non-invasive physical tool, LIPUS is used in rehabilitation, and its role in the field of anti-inflammation is demonstrated via modulating inflammatory responses [19, 20]. LIPUS treatment improves cardiac dysfunction and reduces myocardial fibrosis caused by myocardial infarction, hypertensive heart disease, and viral myocarditis [21–23]. LIPUS can also orchestrate CD4^+^ T-cell immunological status via special mechanical stimulation to activate related pathways. Whether LIPUS ameliorates ICIs myocardial injury by regulating CD4^+^ T-cell differentiation needs further study.

Accordingly, based on the unique therapeutic modality of LIPUS on inflammatory responses, we aimed to determine whether LIPUS improved the myocardial inflammatory responses and cardiac dysfunction associated with ICIs, and if so, explore the intrinsic signaling pathways and molecular mechanisms thereof.

## Materials and methods

### Experimental organisms

Male A/J mice (6–8 weeks of age) were purchased from Beijing Vital River Laboratory Animal Technology (Beijing, China). All animal care and use protocols were in accordance with the Principles of Animal Care provided by the National Society for Medical Research and the Guide for the Care and Use of Laboratory Animals (Institute of Laboratory Animal Resources, NIH). Further, all animal studies conformed to the protocols approved by the Research Ethics Committee of the Second Affiliated Hospital of Harbin Medical University, China (Approval No. SYDW2021-098). To obtain an in vivo model of ICI-related myocarditis, male mice were intraperitoneally treated with an InVivoPlus anti-mouse PD-1 inhibitor (Bioxcell, Lebanon, NH USA) at a concentration of 5 mg·kg^−1^ on days 1, 7, and 14 (in a 21-day cycle).

### LIPUS therapy

For LIPUS treatment, ultrasound equipment from SXULTRASONIC was used with modified therapeutic radiation parameters according to the manufacturer's protocol. During each session, LIPUS stimulation was applied for 20 min at a frequency of 1.0 MHz, duty cycle of 20%, and power of 0.25 W cm^−2^ [22, 24]. LIPUS was performed under isoflurane anesthesia using an ultrasound instrument (Vivid E9; GE Healthcare, Chicago, IL, USA)-guided application to the heart via the chest wall of mice for 20 min daily. Animals in the treatment group received LIPUS every two days for four weeks; in the no-LIPUS group, only anesthesia was administered.

### Echocardiographic evaluation

A high-resolution in vivo imaging system (Vivid E9) was used to observe the echocardiographic parameters and assess cardiac function. Left ventricular internal diameter, diastole (LVID_d_) and left ventricular internal diameter, systole (LVID_s_) were measured by long‐axis views of M‐mode tracings from the anterior to posterior left ventricular wall. The values of the ejection fraction (EF) and fractional shortening (FS) were calculated based on long-axis M-mode measurements from the average of three independent cardiac cycles. All the operations were executed by an experienced physician who was blinded to the groups.

### Histological analysis

The excised hearts were fixed with 4% formalin for histological and immunohistochemical examinations. Following 24–48 h of dehydration, clearing, and embedding in paraffin wax, sliced Sects. (4 mm) were stained with haematoxylin and eosin and Masson’s trichrome and cardiac sections were dewaxed, subjected to antigen retrieval, and incubated with anti-IL17A antibody and anti-FOXp3 antibody (Abcam, Cambridge, UK), followed by incubation with a secondary antibody, and then analyzed using Image J (NIH V1.8.0.112).

### Immunofluorescence

Heart tissues were embedded at the optimal cutting temperature, and sectioned to a thickness of 5 µm. Sections were then stained with anti‐CD3 and anti‐CD4 primary antibodies at 4 °C overnight and then with FITC‐conjugated goat anti‐rabbit IgG at (20–25 °C) for 1 h.

### RNA isolation and quantitative real-time PCR (RT-qPCR)

Total RNA from cultures or sorted CD4^+^ T-cells was isolated using a RNeasy Isolation Kit (Tiangen Biotech, Beijing, China), and then the miRcute Plus miRNA First-Strand cDNA Synthesis Kit (Tiangen Biotech) was used to reverse transcribe RNA to cDNA. Cellular total RNA was extracted using TRIzol reagent (Invitrogen, Carlsbad, CA, USA), and mRNA was reverse transcribed using the Transcriptor First Strand cDNA Synthesis Kit (Roche Diagnostics, Risch-Rotkreuz, Switzerland). All operations followed standard protocols. Gene expression was determined relative to GAPDH and fold change via the 2^−ΔΔCT^ threshold cycle method.

### Flow cytometric analysis

Mouse hearts were removed, homogenized, and filtered through a 70 mm nylon mesh in PBS. Heart tissue was digested and filtered to remove extracellular connective tissue debris. They were then digested in a water bath at 37 °C for 20 min. A single-cell suspension was prepped using a cell filter, whereas cardiac T-lymphocytes were isolated by Ficoll-12 low-density gradient centrifugation and resuspended in a complete RPMI-1640 medium (Thermo Fisher Biochemical, Beijing, China) containing 10% fetal bovine serum. Cells were stained with fixed viability dye (Invitrogen) for 10 min at 20–25 °C to remove dead cells and fluorochrome-labelled monoclonal antibodies against surface cell markers. Cells were fixed and permeabilized using Cytofix/Cytoperm (BD Biosciences; Franklin Lakes, NJ, USA) and perm/wash buffer (BD Biosciences), followed by intracellular staining with monoclonal antibodies for 20 min and incubation with anti-CD4-fluorescein isothiocyanate (FITC) (RM4-5; BD Biosciences) and anti-CD45-phycoerythrin (PE)/Cy7 (30-F11; BioLegend; San Diego, CA, USA). Intracellular staining of transcription factors without stimulation was performed using a FOXp3 Fixation/Permeabilization Kit (eBioscience; San Diego, CA, USA). For IL-17A (B27; BD Biosciences) staining, T-cells were stimulated with phorbol 12-myristate 13-acetate (PMA), ionomycin, and GolgiPlug (Sigma-Aldrich, St. Louis, MO, USA) for 4–5 h. The results were acquired using a FACSCanto II system (BD Biosciences) and analyzed using FlowJo *v.*10.

### Enzyme-linked immunosorbent assay (ELISA)

The expression of T-cell-related cytokines IL-17A and IL-10 was measured using ELISA kits (R&D Systems, Minneapolis, MN, USA) according to the manufacturer’s protocol. Serum markers levels, including creatine kinase-MB (CK-MB) and lactate dehydrogenase (LDH), were determined using commercial assay kits (Nanjing Jiancheng BioTech, Nanjing, China) according to the manufacturer’s instructions.

### Western blotting

Total protein samples from cardiac tissues were extracted with lysis buffer (Beyotime Institute of Biotechnology, Shanghai, China); proteins were separated by SDS-PAGE before being transferred onto polyvinylidene difluoride membranes (Millipore; Burlington, MA, USA). The immunoblots were incubated at 4 °C overnight with primary antibodies, including anti-Myh6 (Abcam; Cambridge, UK), anti-Col1 (Abcam), anti-Mst1 (Abcam), anti-TAZ (Abcam), anti-TEAD-1 (Abcam), anti-FOXp3, anti-RORγt (Santa Cruz), and anti-GADPH (Proteintech, Shanghai, China). Secondary antibodies were purchased from Cell Signaling Technology (Danvers, MA, USA). Samples were normalized to GADPH levels.

#### Administration of MST1/2 inhibitor, XMU-MP-1

XMU-MP-1 (cat #22083) was purchased from Cayman Chemicals (Ann Arbor, MI, USA). XMU-MP-1 was dissolved in dimethyl sulfoxide at a concentration of 30 mg mL^−1^, and administered daily via gavage at a dose of 3 mg kg^−1^ d^−1^ for 4 weeks [25].

#### Statistical analysis

Statistical analyzes were performed using SPSS (*v*.23.0; Chicago, IL, USA) and GraphPad Prism *v.*9.0 (GraphPad Software, San Diego, CA, USA). Statistical significance was indicated by *p* < 0.05 based on a Student’s t-test and one-way analysis of variance.

## Results

### PD-1 inhibitor impairment of cardiac function and induction of myocardial inflammatory response

**Fig. 1:**
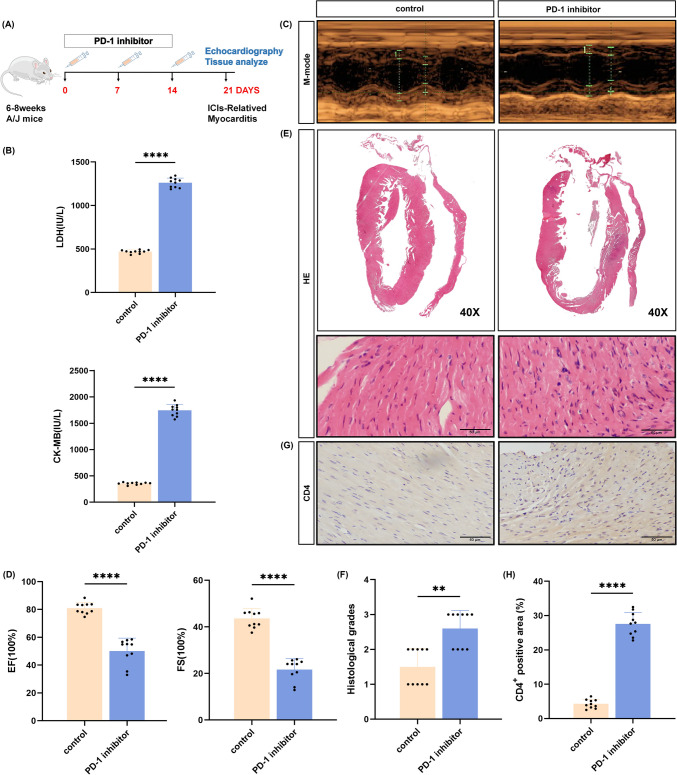
PD-1 inhibitor cause impairment of cardiac function and myocardial inflammatory response. **a** The schedule of injection. A/J mice were randomly divided into 2 groups: control group (control); ICI-related myocarditis group (PD-1 inhibitor).Mice in ICI-related myocarditis group were immunized with PD-1 inhibitor at day 0, 7 and 14.Mice were sacrificed on day 21. **b** Serum levels of creatine kinase-MB (CK-MB), lactate dehydrogenase (LDH) (*n* = 10). **c** Representative image of M-mode echocardiography. **d** Quantifications of ejection fraction (EF) and fraction shortening (FS) (*n* = 10). **e** The heart tissue H&E stain represents the image (scale bar, 50 μm). **f** The H&E images were analyzed based on histological grades (*n* = 10). **g** Immunohistochemistry analyze of CD4 + cell infiltration in cardiac sections (scale bars, 50 μm). **h** The percentages of CD4 + cells were enumerated (*n* = 10). Error bars show mean ± SD.*p < 0.05, **p < 0.01, ***p < 0.001, or ****p < 0.0001

To investigate the role of PD-1 signaling in myocarditis, a mouse model of ICI-related myocarditis was established. Following the molding test experiments, there were no animal deaths recorded during the 21 days. The overall experimental design is shown in Fig. 1a. LDH and CK-MB are common clinical indicators of myocardial injury that can be used to determine disease regression; thus, corresponding levels were measured to assess myocardial damage. Elevated LDH and CK-MB levels were detected in the PD-1 inhibitor-injected mice (Fig. 1b**)**, suggesting cardiac damage in mice with anti-PD-1 myocarditis. Echocardiography was performed on day 21, and M-mode echocardiogram images of the PD-1 inhibitor-treated animals indicated reduced left ventricular wall motion compared to the isotype controls (Fig. 1c). The left ventricular ejection fraction and fractional shortening were also significantly decreased in the PD-1 inhibitor group. Anti-PD-1 treatment led to impaired diastolic and systolic function in animals (Fig. 1d). After echocardiographic assessment of cardiac function, hearts were harvested for HE staining. Similar to the ultrasound findings, a representative histological fraction of anti-PD-1 treated animals showed ventricular dilatation with varying degrees of inflammatory infiltration and cardiomyocyte swelling in the myocardium (Fig. 1e, f). When mice began to exhibit reduced diastolic function of the heart, the cardiac tissue was analzsed using immunohistochemistry, revealing that more CD4^+^ T-cells were located in the cardiomyocytes (Fig. 1g, h). Taken together, these data showed the infiltration of inflammatory cells into myocardial tissue and enhanced T-cell immune responses, suggesting that PD-1 inhibitors can elicit myocardial inflammatory responses and disrupt the balance of the myocardial immune microenvironment.

### LIPUS-ameliorated cardiac inflammation and injury induced by anti-PD-1

**Fig. 2:**
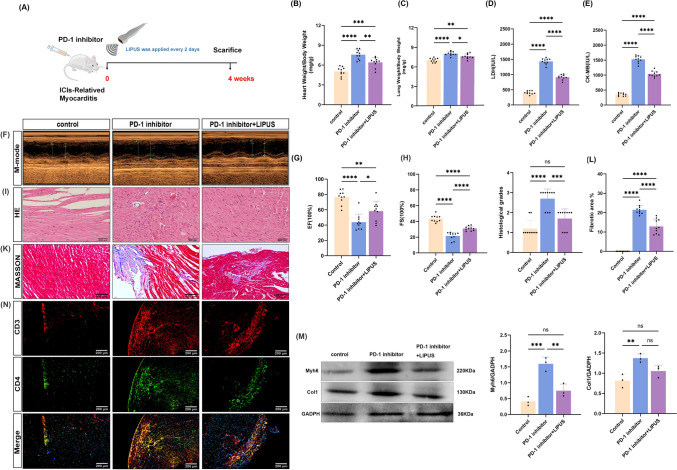
LIPUS Ameliorated Cardiac Inflammation and Injury Induced by PD-1 inhibitor. **a** Experimental protocol. **b**, **c** Ratio of heart weight to body weight;Ratio of lung weight to body weight (*n* = 10). **d**, **e** Serum levels of lactate dehydrogenase (LDH) and creatine kinase-MB (CK-MB) (*n* = 10). **f** Representative image of M-mode echocardiography. **g**, **h** Quantifications of ejection fraction (EF) and fraction shortening (FS) (*n* = 10). **i** The heart tissue H&E stain represents the image (scale bar, 500 μm). **j** The H&E images were analyzed based on histological grades (*n* = 10). **k** Representative image of Masson staining (scale bar, 500 μm). **l** The Masson images were analyzed based on fibrotic area (*n* = 10). **m** Western Blot was performed to assess relative expression of hypertrophy and fibrosis-related genes. **n** Representative images and quantitative analysis of immunofluorescence staining of mice heart sections for CD3 and CD4, (scale bar, 200 μm) (*n* = 10).Error bars show mean ± SD. *p < 0.05, **p < 0.01, ***p < 0.001, or ****p < 0.0001

Whether LIPUS therapy was effective in mice with ICI-related myocarditis was further examined (Fig. 2a). After 4 weeks, the LIPUS treated mice exhibited lower heart weight/body weight (HW/BW) and lung weight/body weight (LW/BW) ratios than the untreated mice (Fig. 2b, c). The index of the myocardial enzyme profile of mice improved but remained higher than that of the control group, with the difference being significant between the PD-1 inhibitor and LIPUS groups (Fig. 2d, e). Echocardiography was used to evaluate the left ventricular function of mice in the two groups after treatment with LIPUS. Compared to the control group, the PD-1 inhibitor group showed obvious heart function injury; however, the mice that received LIPUS treatment displayed stronger recovered cardiac function indices (Fig. 2f–h). Histopathological changes in heart tissue from each group were observed via HE staining. LIPUS treatment significantly reduced inflammatory infiltration caused by the PD-1 inhibitor, and obvious alleviation of cardiac inflammation was observed (Fig. 2i, j). Using Masson staining myocardial fibrosis was found to be reduced by LIPUS treatment, as indicated by reduced collagen deposits in the treated group, thereby suggesting that LIPUS therapy improved fibrotic remodeling (Fig. 2k, l). Western blotting showed that LIPUS treatment reduced the propensity for increased cardiac fibrosis shown by anti-PD-1 treatment; however, cardiac gene expression associated with hypertrophy did not show significant changes (Fig. 2m). Immunofluorescence analysis of heart tissues from mice treated with a PD-1 inhibitor revealed an increase in CD3^+^ and CD4^+^ T-cells. Consistently, the percentages of CD3^+^ and CD4^+^-positive areas were reduced (Fig. 2n) following LIPUS treatment; therefore, LIPUS was concluded to be able to improve the cardiac inflammation and injury induced by anti-PD-1 antibodies.

### LIPUS reduced Th17 cell infiltration and expression of pro-inflammatory cytokines level

**Fig. 3:**
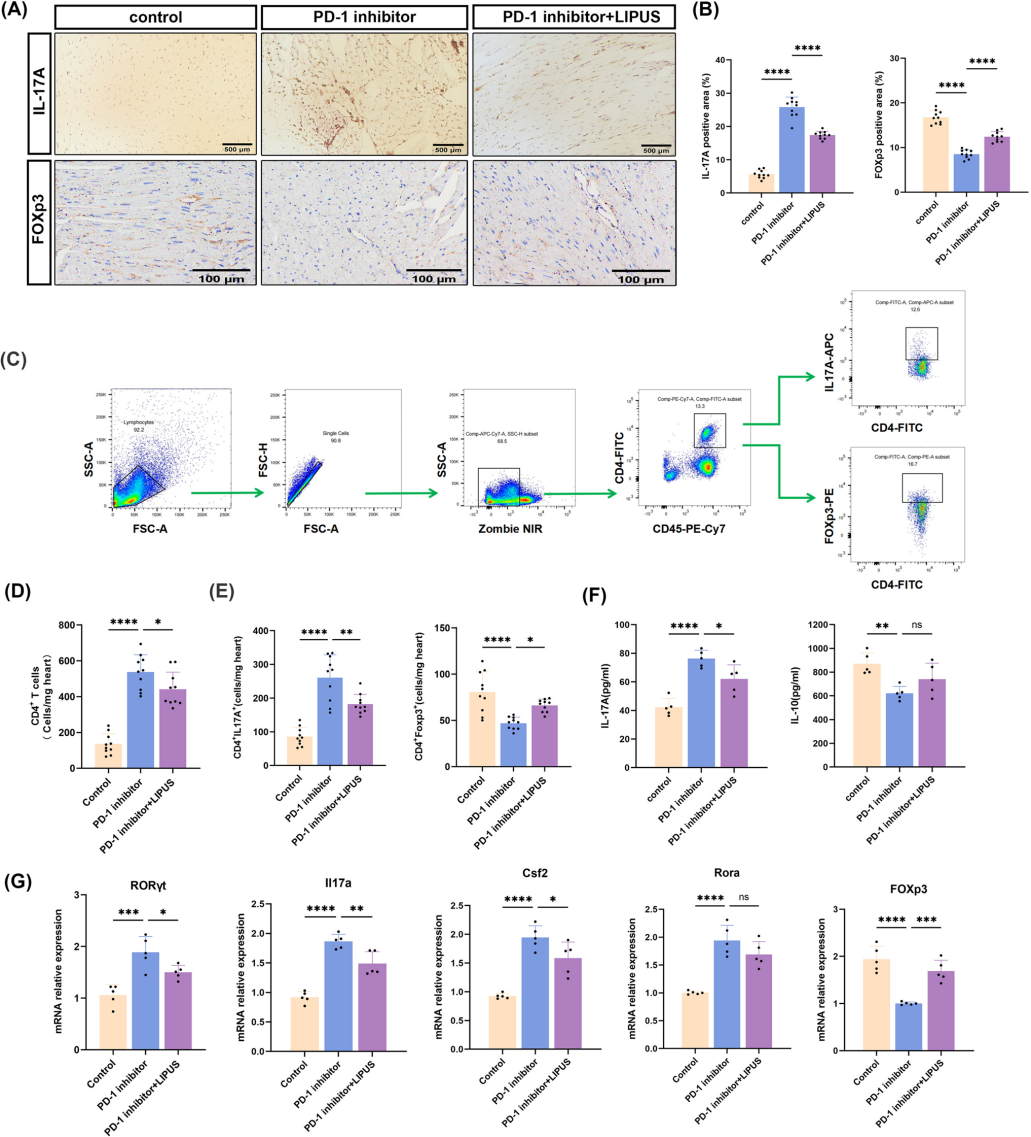
LIPUS reduced cardiac CD4 + T and Th17 responses. **a**–**b** Representative images and quantitative analysis of immunohistochemical analysis of mice heart sections of IL-17A (scale bar, 500 μm) and FOXp3 (scale bar, 100 μm) (*n* = 10). **c** Gating strategy for the flow cytometry experiments. **d** Cardiac infiltrating mononuclear cells were collected from each group for flow cytometry analysis of CD4^+^T cells. **e** Cardiac IL-17A^+^CD4^+^T cells and Foxp3^+^CD4^+^ Tcells for flow cytometry analysis. **f** Levels of the cytokines IL-17A, and IL-10 were measured by ELISA (*n* = 5). **g** Evaluate the expression of RORγt, Il17a, Csf2, Rora and FOXp3 by RT-qPCR; data were normalized to GADPH, (*n* = 5).Error bars show mean ± SD. *p < 0.05, **p < 0.01, ***p < 0.001, or ****p < 0.0001

After demonstrating that LIPUS could alleviate PD-1 inhibitor-induced cardiac dysfunction, CD4^+^ T-cell immune responses following LIPUS therapy on anti-PD-1 inhibitor-induced cardiotoxicity were further examined. Th17 cells are a subset of CD4^+^ T-cells, which themselves are important inflammatory mediators in ICI-related myocarditis, and play a pro-inflammatory role by secreting inflammatory cytokines. [26] To clarify whether LIPUS treatment altered CD4^+^ T-cell differentiation in vivo, cardiac CD4^+^ T-cells were sorted for subsequent analysis. Histopathological analysis showed that LIPUS treatment decreased Th17 pro-inflammatory cell infiltration, as detected by immunostaining for IL-17A in the heart tissue of PD-1 inhibitor mice. In agreement with the phenotypic observations, low FOXp3^+^ regulatory T-cell abundance rebounded (Fig. 3a, b). Flow cytometry of heart T-cells confirmed that the combination of therapeutic LIPUS and anti-PD-1 antibodies significantly reduced the number of CD4^+^ T-cell infiltrating into the heart **(**Fig. 3c, d). Further analysis of CD4^+^ T cell subsets revealed that LIPUS significantly reduced the proportion of Th17 cells and increased the number of Treg cells (Fig. 3e). In addition, ELISA showed increased production of the pro-inflammatory cytokine IL-17A in anti-PD-1 samples; However, IL-10 showed the opposite change whereas LIPUS reversed the levels (Fig. 3f). Accordingly, we detected cytokines related to th17 differentiation, and compared with the PD-1 inhibitor group, transcription levels of RORγt, Il17a, and Csf2 in the LIPUS group were significantly reduced. LIPUS treatment led to the significant induction of FOXp3 mRNA expression under inflammatory conditions (Fig. 3g). These results suggest that LIPUS can prevent Th17 cell differentiation, promote the formation of Tregs, and regulate the transition of the immune microenvironment from a pro-anti-inflammatory state to an anti-inflammatory state in the mouse ICI- related myocardial model.

### Anti-inflammatory effects of LIPUS mediated by altering HIPPO pathway cascade kinase signalling in the myocardium

**Fig. 4:**
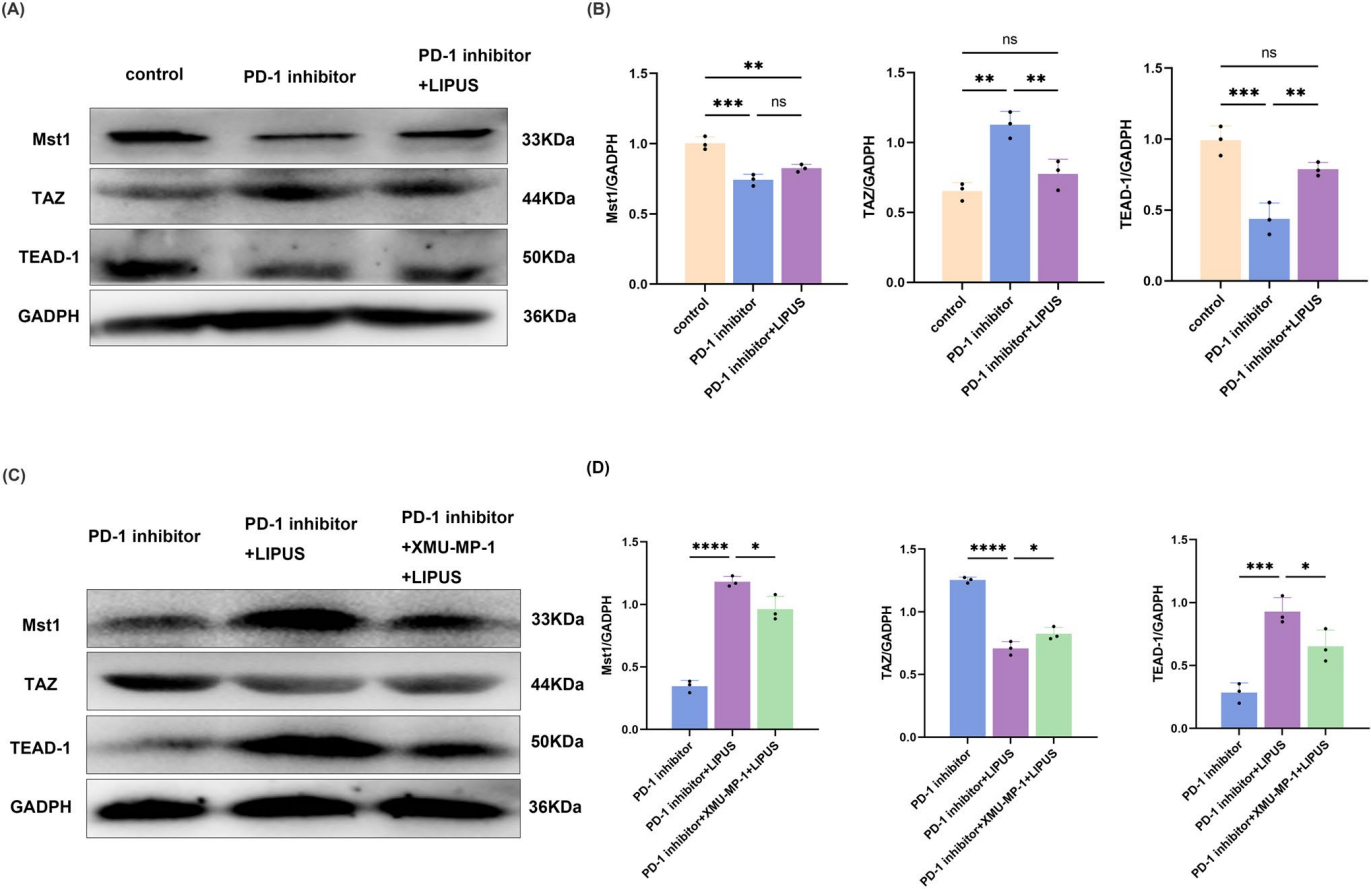
The inflammatory relief of LIPUS is mediated by cascade kinase signaling in the HIPPO pathway. **a**–**d** Representative images and quantitative analysis of Western blotting analysis of HIPPO pathway proteins (Mst1, TAZ and TEAD-1) in different conditions (*n* = 3).(Error bars show mean ± SD. *p < 0.05, **p < 0.01, ***p < 0.001, or ****p < 0.0001

HIPPO signaling maintains the balance between immune restraint and inflammation. The HIPPO signaling pathway was significantly activated in cardiac CD4^+^ T-cells in ICI-related myocarditis (Fig. 4a, b). To confirm the involvement of the HIPPO signaling pathway in the regulation of LIPUS during inflammation relief, the expression of core components of the HIPPO pathway was analzsed, including Mst1, TAZ, and TEAD-1, in myocardial CD4^+^ T-cells. LIPUS treatment increased the expression of Mst1 and TEAD-1 and decreased the expression of TAZ compared with the PD-1 inhibitor group. These results suggested that LIPUS may regulate autoimmune inflammation by downregulating the core kinase Mst1 in the HIPPO pathway and modulate the mutual differentiation of Treg and Th17 cells through altering the interaction between the transcription factors FOXp3 and RORγt by the Mst1–TAZ axis.

### Inhibition of HIPPO signaling pathway diminished therapeutic effects of LIPUS

To directly clarify whether LIPUS alleviates myocardial inflammation and CD4^+^ T-cell differentiation in ICI-related myocarditis by activating the HIPPO pathway, the HIPPO inhibitor XMU-MP-1 was used to constrain the scaffold protein Mst1, followed by LIPUS treatment after the preparation of the ICI-related myocarditis model. Western blot data showed that XMU-MP-1 decreased the cascade of HIPPO pathway kinases as well as the expression levels of Mst1 and TEAD-1 and increased the expression of TAZ in the myocardial tissue of the ICI-related myocarditis model (Fig. 4c, d).

**Fig. 5:**
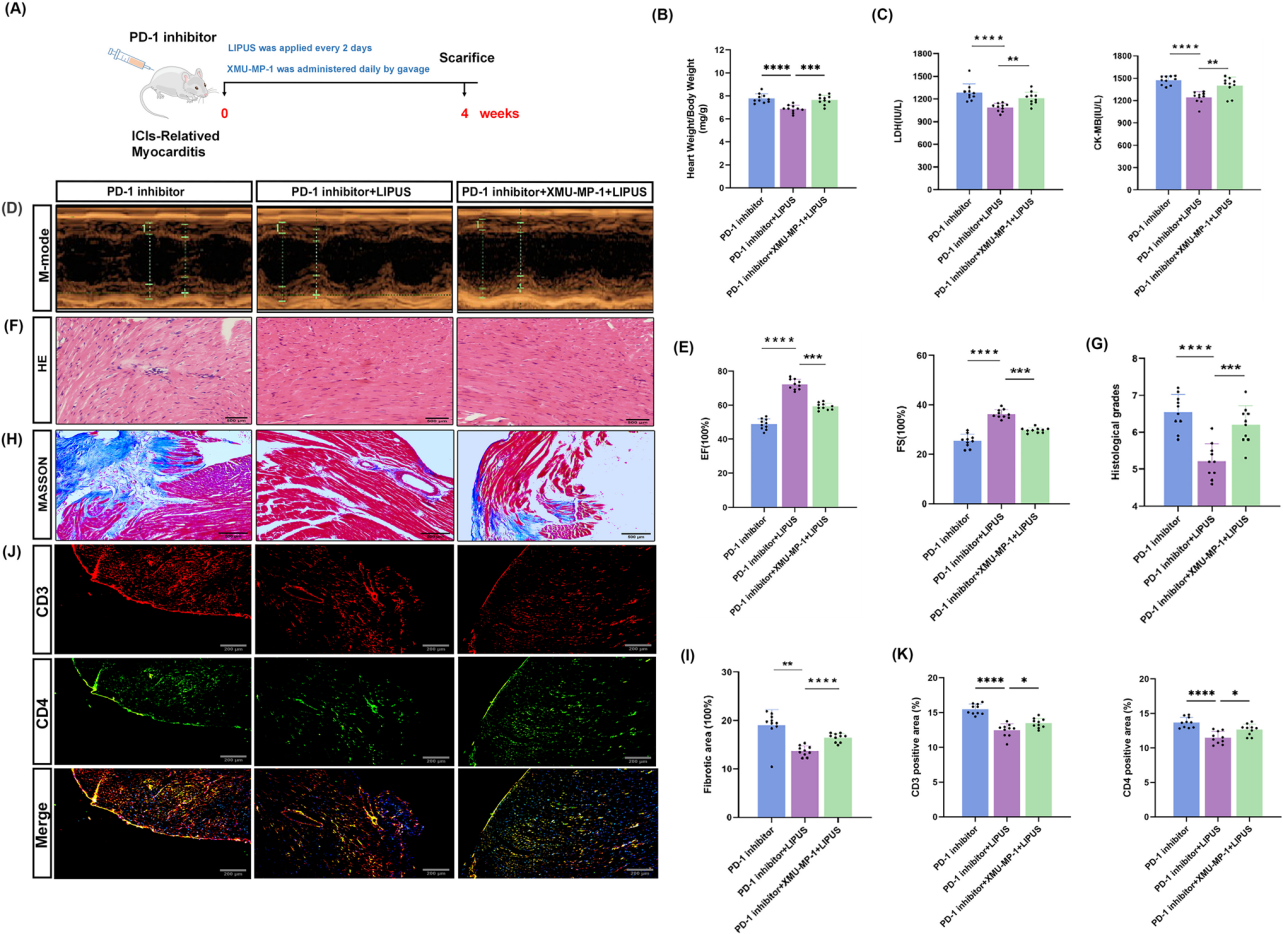
Inhibition of the HIPPO signaling pathway partly diminished the therapeutic effect of LIPUS. **a** Experimental protocol. **b** Ratio of heart weight to body weight. **c** Serum levels of creatine kinase-MB (CK-MB), lactate dehydrogenase (LDH) (*n* = 10). **d** Representative image of M-mode echocardiography. **e** Quantifications of ejection fraction (**e**, **f**) and fraction shortening (FS) (*n* = 10). **f** The heart tissue H&E stain represents the image. (scale bar, 500 μm). **g** The H&E images were analyzed based on histological grades (*n* = 10). **h** Representative image of Masson staining. (scale bar, 500 μm). **i** The Masson images were analyzed based on fibrotic area (*n* = 10). **j** Representative images and quantitative analysis of immunofluorescence staining of mice heart sections for CD3 and CD4, (scale bar, 200 μm) (*n* = 10). **k** Quantifications of immunofluorescence staining. Error bars show mean ± SD. *p < 0.05, **p < 0.01, ***p < 0.001, or ****p < 0.0001

**Fig. 6:**
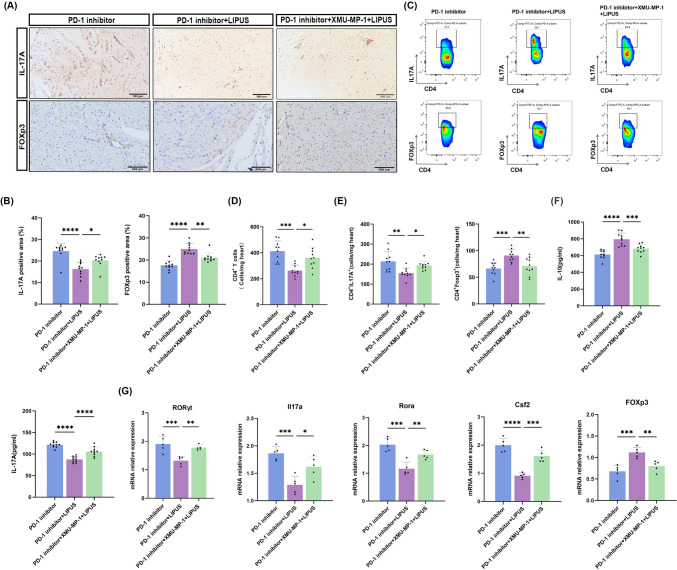
Inhibition of HIPPO signaling pathway partially attenuated the inhibitory effect of LIPUS on CD4^+^T cell differentiation. **a**, **b** epresentative images and quantitative analysis of immunohistochemical analysis of mice heart sections of IL-17A (scale bar, 500 μm) and FOXp3 (scale bar, 500 μm) (*n* = 10). **c** Cardiac infiltrating mononuclear cells were collected from each group for flow cytometry analysis of CD4 + T cells (*n* = 10). **d**, **e** Cardiac IL-17A^+^CD4^+^T cells and Foxp3^+^CD4^+^ T cells for flow cytometry analysis (*n* = 10). **f** Levels of the cytokines IL-17A, and IL-10 were measured by ELISA (*n* = 10). **g** Evaluate the expression of RORγt, Il17a, Csf2, Rora and FOXp3 by RT-qPCR; data were normalized to GADPH, (*n* = 5).Error bars show mean ± SD. *p < 0.05, **p < 0.01, ***p < 0.001, or ****p < 0.0001

A four-week experiment was conducted to explore the effect of the HIPPO pathway inhibitor on general physical indicators in each group (Fig. 5a). XMU-MP-1 administration increased heart weight in LIPUS-treated mice (Fig. 5b). LDH and CK-MB levels were reduced following the application of LIPUS, whereas XMU-MP-1 increased the cardiac enzyme levels again, these values however remained lower than those of the PD-1 inhibitor group (Fig. 5c). In addition, echocardiographic analysis showed that XMU-MP-1 attenuated the LIPUS-induced recovery of cardiac function in mice with ICI-related myocarditis (Fig. 5d, e). PD-1 inhibitors caused ICI-related myocarditis to exhibit high inflammation scores, and LIPUS treatment reduced these. LIPUS treatment also reduced myocardial fibrosis, as indicated by the reduced collagen deposition in the treated group, whereas XMU-MP-1 injection eliminated these positive effects (Fig. 5f, i). Immunofluorescence analysis showed an increase in CD3+ and CD4+ T cells and an increase in the percentage of both CD3+ and CD4+ positive areas in the hearts of XMU-MP-1 treated mice compared with LIPUS-irradiated mouse heart tissues  (Fig. 5j, k). Cardiac tissues were then collected from different groups for immunohistological analyzes. The expressions of IL-17A and FOXp3 showed significant reduction in both inflammatory infiltration and immune response (Fig. 6a, b). HIPPO inhibition significantly attenuated the regulatory effect of LIPUS on CD4^+^ T cell proliferation and differentiation. Compared with the LIPUS group, the proportion of CD4^+^ T cells and Th17 cells was increased, and the proportion of Treg cells was decreased in the HIPPO inhibition group (Fig. 6c–e). Simultaneously, the effects of LIPUS inhibition on cytokine production via CD4^+^ T-cells were partially blocked (Fig. 6f). The effect of XMU-MP-1 on the expression of Th17 cell-related genes was detected. XMU-MP-1 reversed the inhibitory effect of LIPUS on Th17 cell-related genes RORγt, Il17a, Rora and Csf2, whereas reduced the transcription level of FOXp3, and partially restored the inflammatory response (Fig. 6g). XMU-MP-1 weakened the therapeutic effect of LIPUS, and myocardial inflammation in mice was better than that in the PD-1 inhibitor group; therefore, activation of the HIPPO pathway in CD4^+^ T-cells contributes to LIPUS for improving ICI-related myocarditis.

## Discussion

The present study established a mouse model of ICI-related myocarditis and quantified the effects of LIPUS on regulating the pathogenesis of ICI-related myocarditis. First, LIPUS treatment attenuated cardiac immune inflammatory responses and cardiac dysfunction induced by PD-1 inhibitors. Second, LIPUS inhibited the differentiation of CD4^+^ T-cells, reduced the infiltration of Th17 cells into the CD4^+^ T-cell subset in ICI-related myocarditis, and increased the ratio of Treg cells, thereby alleviating inflammatory responses. Third, based on the in vivo experiments, LIPUS regulated the balance of Th17/Treg immune microenvironment to exert its therapeutic effects.

Cancer treatment options have expanded greatly over the past decade. Immunotherapy, wherein ICIs strengthen the body's own immune cell activity against cancer cells, has become prominent [27]; however, an increasing number of investigators are highlighting the characteristics of irAEs which are critical for patient safety. Simultaneously, the toxicity of immunotherapy caused by ICIs limits their therapeutic prospects [28]. Approximately 60–80% of patients treated with ICIs experience irAEs of different grades, including endocrinopathies, colitis, and myocarditis [29].

Adverse cardiovascular events associated with ICI use have been increasingly reported. ICI-related myocarditis is rare, but fatal adverse events characterized by cardiac arrest or fatal arrhythmia are often reported [30]. Patients typically present with chest pain, elevated cardiac troponin levels, or abnormal cardiac imaging. Because of the nonspecific symptomatology and fulminant progression, clinicians should be highly alert to ICI-related myocarditis [31, 32]. In light of this, glucocorticoids combined with a variety of low-dose immunosuppressive agents have received extensive attention; however, their application and risks require further clarification. Safer and more effective treatment options are urgently needed [33]. The myocardial enzyme detection and imaging results of the mice in this study were consistent with those reported in the literature, which also made the results of our preliminary model preparation more reliable.

The pathological features of ICI include the expansion and activation of antigen-driven T-lymphocytes mediated by ICIs, production of excessive inflammatory factors, and balance disruption of the autoimmune environment, leading to the infiltration of T-lymphocytes (primarily CD4^+^ T-cells) and myocyte death in the myocardium [34, 35]. PD-1 is mainly expressed in T-cells, suggesting that they play a crucial role in the development of ICI-related myocarditis [36]. The primary features of ICI-myocarditis are inflammatory damage and fibrosis in the heart, leading to cardiac dysfunction and tissue remodeling. Additionally, the main triggers of ICI-myocarditis factors are activated CD4^+^ T-cells, which direct the differentiation of CD4^+^ T-cells into Th17 effector cells and are vital in establishing the inflammatory process that results in myocardial tissue injury [37]. In the present study, the infiltration of CD4^+^ T-cells into the myocardium of mice increased following the administration of a PD-1 inhibitor, suggesting that the modulation of CD4^+^ T-cells may provide a novel therapeutic direction for ICI-related myocarditis.

LIPUS, which produces sound waves, has been employed as a physical therapy technique [38, 39] that results in a cascade of biochemical events, not adverse effects on the cells themselves [40, 41]. LIPUS has been primarily used for rehabilitation [42]; however, with increasing research its potential in mediating inflammation has been gradually revealed. It is being widely considered as a novel, non-invasive approach for treatment of cardiovascular disease [43]. LIPUS treatment improves ischemia-induced cardiac dysfunction and angiotensin II (AngII)-induced myocardial fibrosis [44]. In addition, LIPUS has been found to ameliorate left ventricular remodeling following myocardial infarction in mice [45, 46].

Lymphocytes have been shown to be sensitive to ultrasonic mechanical stress, and the mechanical channels of cytokines in lymphocytes can be regulated by mechanical waves, which play an important role in the inflammatory process [47, 48]. We attempted to use LIPUS as a treatment for ICI-related myocarditis caused by PD-1 inhibitors. A mouse model of ICI-related myocarditis was developed, and the condition of murine myocarditis was documented. Following cycles of PD-1 inhibitor treatment, systolic and diastolic functions of the heart were significantly reduced, and myocardial inflammatory damage was observed, which is notably consistent with previous findings [49]. An M-mode echocardiography was used to obtain Left Ventricular Ejection Fraction (LVEF) values. A previous study found that LIPUS improved cardiac diastolic dysfunction in obese diabetic mice [50]. Histopathological data demonstrated that LIPUS treatment improved the cardiac structural and hemodynamic changes stimulated by PD-1 inhibitors and reduced myocardial fibrosis. LIPUS is thereby suggested to play an important role in the repair of heart damage. A mouse model of ICI-related myocarditis mimicked the pathogenesis of myocarditis, with peak cardiac inflammation occurring between 14 and 21 d, and was characterized by the infiltration of heart-specific CD4^+^ T-cells into the myocardium [51]. The data presented here showed that Th17/Treg imbalance and increased pro-inflammatory factor expression occurred in cardiac-infiltrating CD4^+^ T-cells following PD-1 inhibitor injection cycles. After treatment, LIPUS reversed the imbalance of Th17 and Treg cells by altering the interactions between transcription factors FOXp3 and RORγt and reducing the expression of a series of ICI-related myocarditis inflammatory factors, thereby inhibiting the development of myocardial inflammation suggesting that LIPUS may have a positive regulatory effect on inflammation in ICI-related myocarditis.

The downstream HIPPO signaling pathway responds to extracellular signals; plays a key role in tissue homeostasis, organ regeneration, and tumorigenesis [52]; and is involved in regulating the differentiation and function of immune cells, independent of its classical regulation [53]. Intercellular communication has been identified as an important signal in HIPPO pathway regulation, in which mechanical stimulation also serves as a potent regulator [54, 55]. Multiple core components of the HIPPO pathway are involved in the regulation of immune responses [56]. MST1 enhances Foxo1/3 stability in CD4^+^ T-cells through direct phosphorylation as well as promoting FOXp3 expression and Treg cell development by attenuating TCR-induced AKT activation in peripheral blood T-cells [57]. It was recently found that Mst1/2–TAZ signaling inhibits the development of inflammatory Th17 cells and enhances the differentiation of immunosuppressive Treg cells, which is critical for preventing autoimmune disease development and maintaining immune homeostasis [58]. We found that ICI-related myocarditis reduced Mst1 kinase activity and activated TAZ, acting as a co-activator of RORγt to promote Th17 differentiation and inhibit Treg cell development. This suggests that HIPPO signaling activation and TEAD negatively regulate TAZ-mediated Th17 differentiation. Similar experiments have shown that TEAD1 has a higher affinity for TAZ than ROR or FOXp3 and can destroy the interaction between TAZ and ROγt or FOXp3. Moreover, TEAD1 significantly decreased TAZ- or RORγt-mediated activity of the Th17. Comparatively, strong TEAD1 expression sequestered TAZ from RORγt and FOXp3 to positively promote Treg cell differentiation [59, 60]. LIPUS regulates the expression of key kinases of the HIPPO pathway through mechanical acoustic pressure changes in ICI-related myocarditis, improving the imbalance of the immune microenvironment between Th17 and Treg cells and alleviating myocardial inflammatory injury. LIPUS therapy, via HIPPO pathway activation, is proposed to alleviate ICI-induced myocardial inflammation.

Several limitations from the present study should be acknowledged. First, the effects of longer LIPUS treatments (> four weeks) were not investigated. In addition, LIPUS irradiation was applied following the successful establishment of the ICI-related myocarditis model; thus, the combination of LIPUS irradiation and anti-PD-1 treatment should be explored in future studies. The present study aimed to explore the autoimmune response during LIPUS treatment of ICI-related myocarditis, which is primarily mediated by CD4^+^ T-cells; thus, CD4^+^ T cells were selected for the present analyzes. The treatment of CD4^+^ T cells with LIPUS and its effect on other immune cells was not explored. Reports have demonstrated the critical role of CD8^+^ T cells in the disease pathophysiology. Administration of CD8^+^ T-cell-depleting antibodies conferred a significant survival benefit [61]. Whether LIPUS can alleviate the myocardial inflammatory effects of ICI by inhibiting CD8^+^T cells should be the focus of our future research. Accumulating evidence suggests that bystander activation of T cells independent of TCR-specific recognition occurs in the inflammatory environment of autoimmune diseases. In further studies, it should focus on the fine-tuning mechanism and interaction of bystander-activated CD4^+^T cells in the development of ICI-related myocarditis [62, 63]. Lastly, Mst1 conditional knock-out (KO) mice were not used in the in vivo study; they should be used to confirm to confirm the effect of LIPUS on ICI-related myocarditis.

However, clinical trials of LIPUS for heart disease are not yet available; therefore, a large prospective cohort study should be conducted first. There are several points should be emphasized. Firstly, because of species differences between animals and humans, it is necessary to screen parameters that are best suited to humans. Second, the appropriate period of treatment should be explored. In conclusion, anti-PD-1 treatment disrupts myocardial immune homeostasis and induces inflammatory responses. Additionally, it was found that LIPUS treatment ameliorated ICI-induced inflammatory myocardial injury, reduced CD4^+^ T-cell infiltration into the myocardium, inhibited Th17 differentiation, activated the transcription factor FOXp3, and promoted Treg differentiation. This process involves mechanotransduction and regulation of the downstream HIPPO pathway; therefore, LIPUS therapy may represent a promising, non-invasive treatment strategy for ICI-associated myocarditis.

### Supplementary Information

Below is the link to the electronic supplementary material.

Supplementary file1 (DOCX 12 KB)

Supplementary file2 (DOCX 12 KB)

## Data Availability

All data generated or analyzed during this study are included in this published article.
