# Supplementary material for: Protective effect of low‐intensity pulsed ultrasound on immune checkpoint inhibitor-related myocarditis via fine-tuning CD4+ T-cell differentiation
Source: Cancer Immunol Immunother. 2024 Jan 18;73(1):15. doi: 10.1007/s00262-023-03590-5 (PMC10796578; doi:10.1007/s00262-023-03590-5)
Supplement: Supplementary file 1 — Supplementary file1 (DOCX 12 KB) [file 262_2023_3590_MOESM1_ESM.docx]

Table S1.  **KEY RESOURCES TABLE**

| **Antibodies** | **catalog numbe**r | **SOURCE** |
| --- | --- | --- |
| Anti-CD3 | 14-0030-82 | Thermo Fisher Scientifific |
| Anti-CD4 | 14-0041-82 | Thermo Fisher Scientifific |
| Anti-IL17A | ab79056 | Abcam |
| Anti-FOXP3 | ab215206 | Abcam |
| Anti-CD4 FITC | 553046 | BD biosciences |
| Anti-CD45 PE/Cy7 | 103113 | Biolegend |
| Anti-FOXp3 PE | 560414 | BD biosciences |
| Anti-IL-17A PE | 562016 | BD biosciences |
| mouse IL10 Quantikine ELISA Kit | M10008 | R&D Systems |
| mouse IL17 Quantikine ELISA Kit | M1700 | R&D Systems |
