# Supplementary material for: Protective effect of low‐intensity pulsed ultrasound on immune checkpoint inhibitor-related myocarditis via fine-tuning CD4+ T-cell differentiation
Source: Cancer Immunol Immunother. 2024 Jan 18;73(1):15. doi: 10.1007/s00262-023-03590-5 (PMC10796578; doi:10.1007/s00262-023-03590-5)
Supplement: Supplementary file 2 — Supplementary file2 (DOCX 12 KB) [file 262_2023_3590_MOESM2_ESM.docx]

Table S2. **List of mouse primer pairs used for RT-qPCR analysis**

| PCR | Primer | Sequence |
| --- | --- | --- |
| Rora | Forward | 5’-TCTCCCTGCGCTCTCCGCAC-3’ |
| Rora | Reverse | 5’-TCCACAGATCTTGCATGGA-3’ |
| FOXp3 | Forward | 5’-CACCTATGCCACCCTTATCCG-3’ |
| FOXp3 | Reverse | 5’-CATGCGAGTAAACCAATGGTAGA-3’ |
| Il17a | Forward | 5’-GCTCCAGAAGGCCCTCAG-3’ |
| Il17a | Reverse | 5’-CTTTCCCTCCGCATTGACA-3 |
| Csf2 | Forward | 5’-TTTACTTTTCCTGGGCATTG-3’ |
| Csf2 | Reverse | 5’-TAGCTGGCTGTCATGTTCAA-3’ |
| Gapdh | Forward | 5’-CATCTTCTTGTGCAGTGCCA-3’ |
| Gapdh | Reverse | 5’-CGGCCAAATCCGTTCAC-3’ |
